# Trends and socioeconomic disparities in diabetes prevalence and quality of care among Israeli children; 2011-2018

**DOI:** 10.1186/s13584-020-00399-w

**Published:** 2020-08-20

**Authors:** Yiska Loewenberg Weisband, Michal Krieger, Ronit Calderon-Margalit, Orly Manor

**Affiliations:** 1 Braun School of Public Health and Community Medicine, Hebrew University-Hadassah, Jerusalem, Israel; 2 National Program for Quality Indicators in Community Healthcare, Jerusalem, Israel

**Keywords:** Socioeconomic disparities, Pediatric diabetes, Diabetes prevalence, Quality of care

## Abstract

**Background:**

Despite Israel’s universal health coverage, disparities in health services provision may still exist. We aimed to assess socioeconomic disparities in diabetes prevalence and quality of care among Israeli children, and to assess whether these changed over time.

**Methods:**

We used repeated cross-sectional analyses in the setting of the National Program for Quality Indicators in Community Healthcare. The data were derived from electronic medical records from Israel’s four health maintenance organizations. The study population included all Israeli children aged 2–17 years in 2011–2018 (2018: *N* = 2,404,856). Socio-economic position (SEP) was measured using Central Bureau of Statistics data further updated by a private company (Points Business Mapping Ltd), and grouped into 4 categories, ranging from 1 (lowest) to 4 (highest). We used logistic regression to assess the association of SEP with diabetes prevalence, diabetes clinic visits, hemoglobin A1C (HbA1C) testing, and poor glycemic control (HbA1c > 9%), and assessed whether these changed over time.

**Results:**

Diabetes prevalence increased with age and SEP, with a total of 3019 children with diabetes. SEP was positively associated with visiting a specialized diabetes clinic (age and sex adjusted Odds Ratio (aOR_SEP 4 vs. 1_ 2.45, 95% Confidence Interval (CI) 1.67–3.69)). Although children in higher SEPs were less likely to undergo HbA1c testing (aOR_SEP 4 vs. 1_ 0.54, 95% CI 0.40–0.72), they were also less likely to have poor glycemic control (aOR_SEP 4 vs. 1_ 0.25, 95% CI 0.18–0.34). Disparities were especially apparent among children aged 2–9 (6.5% poor glycemic control in SEP 4 vs. 38.2% in SEP 1). Poor glycemic control decreased over time, from 44.0% in 2011 to 34.1% in 2018.

**Conclusions:**

While poor glycemic control rates among children have improved, they remain high compared to rates in adults. Additionally, substantial socioeconomic gaps remain. It is eminent to study the causes of these disparities and develop policies to improve care provided to children in the lower SEP levels, to promote health equity.

## Introduction

Several multi-national registries have documented increasing rates of type 1 diabetes mellitus (T1DM) in children [1, 2]. Most European countries have shown an increase in pediatric T1DM incidence rates [3]. However, some countries with high incidence rates, including Sweden, Norway and Finland, have seen a plateau in incidence rates [4–6]. In addition, large variability exists in T1DM prevalence and incidence between countries [2].

Good glycemic control is crucial in children with T1DM, as this reduces the risk of long-term complications, including end-stage renal disease, blindness, and lower-limb amputation [7]. Poor glycemic control has been reported to be associated with lower socio-economic position (SEP) in studies from the US and Europe [7–9]. It is unclear whether this association stems from problems in access to care, or from other factors associated with low SEP.

Routine care for the management of diabetes in children and adolescents differs from the care provided to adults with diabetes, since the epidemiology, pathophysiology, developmental considerations, and response to therapy in childhood diabetes are different from diabetes in adults [10]. Pediatric diabetes care should be provided by a multidisciplinary team trained in childhood diabetes management. This care should include diabetes self-management education, psycho-social therapy and medical nutrition [10].

In Israel, all residents have universal health coverage. A previous Israeli study found that in adults lower socioeconomic position was associated with poor glycemic control [11]. An additional study found higher rates of poor glycemic control in Arab children compared to Jewish children [12]. However, it is unclear whether there is an association between SEP and diabetes care on a population-level, in a country with health care for all. We therefore aimed to assess socioeconomic disparities in diabetes prevalence and quality of care among children in Israel, and to assess whether diabetes prevalence and quality of care changed over time.

## Methods

All Israeli residents are entitled to a standardized basket of medical services, which are provided by four health maintenance organizations (HMOs), under the National Health Insurance Law. All residents must be associated with an HMO of their choice, and are free to transfer between HMOs [13]. The Israeli Quality Indicators in Community Healthcare (QICH) program was created to monitor and evaluate the quality of services provided by the four HMOs. The QICH program was initiated in 2002 and was adopted as a national program in 2004 [14]. Our study used QICH data to assess the prevalence of diabetes among Israeli children and the quality of pediatric diabetes care.

### Population

The study population included all Israeli residents aged 2–17 years in 2011–2018. The data includes children with complete membership for a given year. Children who switched from one HMO to another during the calendar year, those deceased during the year, and those residing abroad for more than two years were not included. Transfers between HMOs were infrequent, with less than 2% of the population transferring annually, [15] and the other categories were negligible. Information regarding diabetes prevalence was available for children ages 2–17. In total, 2,404,856 children were included for 2018, of which 3019 were children with diabetes.

### Data

The QICH program received anonymized and aggregated data for 2011–2018, originating from patient electronic medical records from the four HMOs. Our study consisted of physician visits, visits to pediatric diabetes centers, and laboratory testing. We used four indicators regarding childhood diabetes. The first assessed the prevalence of diabetes among children aged 2–17 years. Children were considered to have diabetes in a specific calendar year if they purchased at least three prescriptions for insulin medications in different months, in the previous year. According to the latest report of Israel’s National Registry of Insulin-Dependent (Type 1) Diabetes in ages 0–17, 92.5% of new diabetes cases in children were type 1, and only 5% were type 2 [16]. The national registry defines new cases of diabetes, according to the date of the first insulin injection. In addition, most Israeli children with type 2 diabetes are not treated with insulin. (personal communication) Therefore, our definition of diabetes refers to type 1 diabetes. The following variables assessed the quality of care provided to children with diabetes, and were only assessed among children who were defined as having diabetes in the previous measure. The first assessed the proportion of children with diabetes who visited a pediatric diabetes clinic at least once in the past year. The two additional indicators pertain to hemoglobin A1c (HbA1c) testing. We assessed the proportion of children with diabetes that were tested for HbA1c, as well as the proportion of children who had poor glycemic control (HbA1c > 9%).

### Socioeconomic position (SEP)

SEP was established using small statistical areas (SSA) from the 2008 Israeli census. SSAs include 3000–4000 people, and are created in a way that maintains homogeneity in terms of the socio-demographic distribution of the population. The SSAs were categorized into 20 groups, ranging from 1 (lowest) -20 (highest) [17]. Israel’s central bureau of statistics (CBS) used information regarding housing conditions, education levels, daily functioning, employment, and household income to obtain a measure of SEP for each SSA. As the most recent available data from the CBS was from 2008, we used data updated by the POINTS Location Intelligence Company to improve the timeliness of the SEP measure [18]. The POINTS Company used more recent information on education, income, living conditions, and demographic data to update the SSAs received from the census data, and grouped them into 10 SEP categories, ranging from 1 (lowest) to 10 (highest). We furthered grouped these into 4 categories, for ease of reporting, as follows: SEP 1–3: I, 4–5: II, 6–7: III, 8–10: IV. SEP was available from 2014.

### Statistical analysis

We report the age and socio-economic distribution of children and adolescents with diabetes as well as the general population of children and adolescents aged 2–17. Data regarding children in the general population included children aged 2–19, due to limitations in data availability. We report the prevalence of diabetes, diabetes clinic visits, HbA1c testing and HbA1c over 9%, stratified by age and SEP in 2018. We used univariable logistic regression to assess whether the association between prevalence of diabetes, diabetes clinic visits, HbA1c testing and HbA1c over 9%, and SEP (as a continuous variable) displayed a linear trend.

We used multivariable logistic regression to assess the association between SEP and diabetes prevalence, diabetes clinic visits, HbA1c testing and HbA1c over 9%, adjusting for age, and sex. Finally, we evaluated whether prevalence of diabetes, diabetes clinic visits, HbA1c testing and HbA1c over 9% changed over time, overall, stratified by age group and stratified by SEP, using logistic regression. Trends over time stratified by SEP were restricted to 2014–2018, as SEP was only available from 2014. We based statistical significance on a *p*-value of 0.05, and reported 95% confidence intervals. Analysis was performed using Stata V.14.0 (StataCorp, College Station, Texas, USA).

## Results

Among children with diabetes, nearly 80% were 10 years of age or older, while only 2.5% were between ages 2 and 4 (Table 1). The distribution of SEP was similar among children with diabetes and in the general population. SEP was missing for 4.9% of children with diabetes, and 4.7% of children in the general population.

**Table 1:** Distribution of age, sex and socio-economic position among 2–17-year-old children with diabetes, and the general population, in Israel, 2018

	Children with Diabetes		General Population	
	N	%	*N*	%
Age				
2–4	77	2.6	504,819	21.0
5–9	538	17.8	789,602	32.8
10–14	1294	42.9	719,259	29.9
15–17^*^	1110	36.8	391,176	16.3
Sex				
Male	1522	50.4	1,234,005	51.3
Female	1497	49.6	1,170,851	48.7
SEP^**^				
1	750	24.8	640,800	26.6
2	850	28.2	651,256	27.1
3	812	26.9	666,441	27.7
4	458	15.2	333,508	13.9
Total	3019		2,404,856	

^*^ Among the general population this included children ages 15–19

^**^ SEP – socio economic position was missing for 149 children with diabetes (4.9%), and 112,851 children in the general population (4.7%)

Diabetes prevalence increased as SEP increased, among children aged 15 and older, while there was no statistically significant increase among younger children (Table 2). The prevalence of diabetes clinic visits declined with age, while the prevalence of diabetes clinic visits increased as SEP increased (linear trend OR: 1.28; 95% CI 1.15–1.43). HbA1c testing was highest among children in the lowest SEP, for all age groups, and decreased as SEP increased (overall linear trend OR: 0.76; 95% CI:0.70–0.82). We found substantial SEP disparities in children with HbA1c over 9%. While only 6.5% of children aged 2–9 years in SEP 4 had poor glycemic control, 38.1% of children in SEP 1 had HbA1c over 9%. In children aged 10–14 and 15–17, the disparities were less dramatic, but the prevalence of poor glycemic control in children in SEP 1 remained over twice that in SEP 4 (53.3 vs. 19.1 and 41.8 vs. 14.4 respectively) (linear trend across all SEP OR: 0.59; 95% CI 0.54–0.65).

**Table 2:** Prevalence of diabetes, diabetes clinic visits, HbA1c testing and HbA1c over 9% by SEP and age, 2018

	Total	SEP 1	SEP 2	SEP 3	SEP 4	OR^*^ (linear trend)	*P*-value (trend)
		%	%	%	%		
**Diabetes Prevalence**							
**Age groups**							
2–9	0.05	0.04	0.05	0.04	0.06	1.05	0.198
10–14	0.18	0.17	0.18	0.18	0.18	1.02	0.409
15–17	0.28	0.24	0.30	0.29	0.32	**1.04**	**0.004**
Total (*n* = 2,267,785)	0.13	0.12	0.13	0.12	0.14	**1.04**	**0.045**
**Diabetes clinic visits**							
**Age groups**							
2–9	90.89	91.10	89.27	91.19	92.16	1.06	0.674
10–14	86.48	83.48	87.39	87.47	90.67	**1.21**	**0.023**
15–17	84.86	78.23	84.26	85.71	94.48	**1.47**	**< 0.001**
Total (*n* = 3019)	86.78	83.07	86.59	87.56	92.36	**1.28**	**< 0.001**
HbA1c **testing**							
**Age groups**							
2–9	71.06	80.82	70.06	65.41	60.78	**0.73**	**< 0.001**
10–14	77.20	81.08	81.09	72.70	67.88	**0.77**	**< 0.001**
15–17	81.26	85.61	83.02	79.59	72.39	**0.77**	**< 0.001**
Total (*n* = 3019)	77.44	82.67	79.53	73.77	67.90	**0.76**	**< 0.001**
HbA1c **over 9%**							
**Age groups**							
2–9	24.49	38.14	26.61	11.54	6.45	**0.48**	**< 0.001**
10–14	38.94	53.33	40.99	26.05	19.08	**0.57**	**< 0.001**
15–17	33.26	41.81	36.80	26.07	14.41	**0.65**	**< 0.001**
Total (*n* = 2338)	34.05	46.13	36.69	23.54	14.79	**0.59**	**< 0.001**

^*^We used univariable logistic regression to assess whether the association between prevalence of diabetes, diabetes clinic visits, HbA1C testing and HbA1C over 9%, and SEP (as a continuous variable) displayed a linear trend. OR reflects a one-unit change in SEP. Results in bold indicate statistical significance at the 0.05 level

Controlling for age and sex, diabetes prevalence was positively associated with SEP (Table 3). Children in the highest SEP (SEP 4) had higher odds of visiting a specialized diabetes clinic compared to children in the lowest SEP (SEP 1)(aOR_SEP 4 vs 1_ 2.45, 95% CI 1.67–3.69). Children in the highest SEP had lower odds of undergoing HbA1c testing compared to those in the lowest SEP (AOR_SEP 4 vs 1_ 0.45, 95% CI 0.34–0.59). However, among those who were tested, the odds of having poor glycemic control (HbA1C > 9%) was significantly lower in the highest SEP compared to the lowest SEP (AOR_SEP 4 vs 1_ 0.20, 95% CI 0.14–0.28).

**Table 3:** Multivariable models assessing odds of diabetes prevalence, diabetes clinic visits, HbA1C testing and HbA1c > 9%, among Israeli children aged 2–17, 2018

	Diabetes Prevalence			Diabetes clinic visits			HbA1c testing			HbA1c over 9%		
	OR^1^	95% CI	*p*-value	OR^1^	95% CI	*P*-value	OR^1^	95% CI	*p*-value	OR^1^	95% CI	*p*-value
**Age**												
2–9	Ref			Ref			Ref			Ref		
10–14	**3.79**	**3.44–4.18**	**< 0.001**	**0.68**	**0.49–0.94**	**0.020**	**1.41**	**1.13–1.76**	**0.003**	**2.08**	**1.59–2.74**	**< 0.001**
15–19	**5.98**	**5.40–6.62**	**< 0.001**	**0.56**	**0.40–0.78**	**< 0.001**	**1.85**	**1.46–2.35**	**< 0.001**	**1.64**	**1.24–2.17**	**< 0.001**
**Sex**												
Male	Ref			Ref						Ref		
Female	1.05	0.97–1.13	0.222	0.88	0.71–1.10	0.270	1.09	0.91–1.30	0.357	1.08	0.90–1.30	0.432
**SEP**												
1	Ref			Ref			Ref			Ref		
2	**1.15**	**1.04–1.27**	**0.005**	**1.32**	**1.00–1.73**	**0.050**	0.81	0.63–1.05	0.11	**0.67**	**0.54–0.84**	**< 0.001**
3	1.10	1.00–1.22	0.057	**1.44**	**1.08–1.91**	**0.012**	**0.59**	**0.46–0.75**	**< 0.001**	**0.35**	**0.27–0.45**	**< 0.001**
4	**1.21**	**1.08–1.36**	**0.001**	**2.45**	**1.67–3.69**	**< 0.001**	**0.45**	**0.34–0.59**	**< 0.001**	**0.20**	**0.14–0.28**	**< 0.001**

^1^Models adjusted for age, sex, and SEP. Results in bold indicate statistical significance at the 0.05 level

**Fig. 1:**
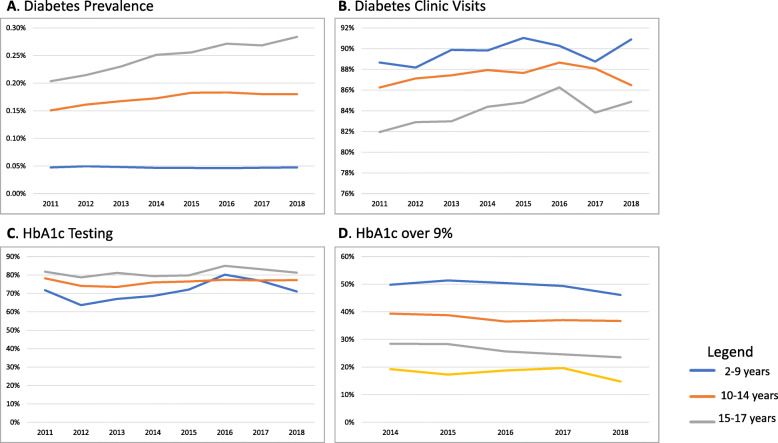
Diabetes prevalence, clinic visits, HbA1c testing and HbA1c over 9% rates over time, by age groups

Diabetes prevalence rates remained stable over time among children aged 14 and younger, however there was a slight increase among children aged 15–17 (P_trend_ < 0.001) (Fig. 1a). There was no statistically significant change in the overall prevalence of diabetes clinic visits (P_trend_ = 0.51) (Fig. 1b). HbA1c testing increased slightly over time (P_trend_ = 0.02) (Fig. 1c). We found a substantial reduction in the prevalence of children with HbA1c over 9% over time, across all age groups (P_trend_ < 0.001) (Fig. 1d). Throughout the entire time period we examined, children aged 10–14 had the highest prevalence of poor glycemic control.

**Fig. 2:**
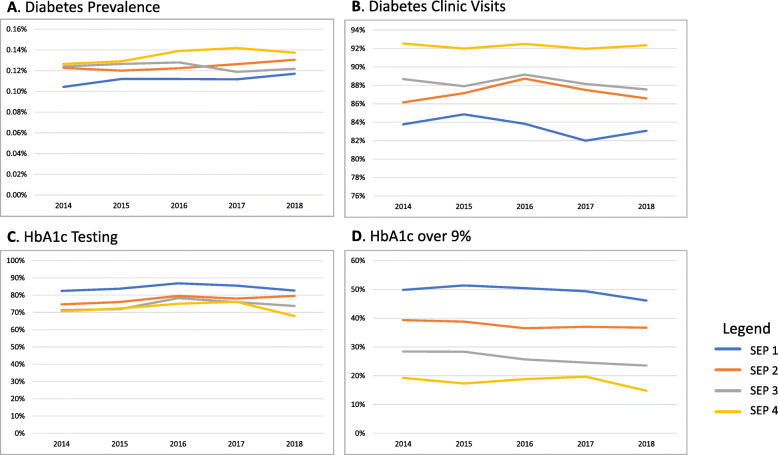
Diabetes prevalence, clinic visits, HbA1c testing and HbA1c over 9% rates over time, by socio-economic position (SEP). ^*^Trends over time stratified by SEP are limited to 2014–2018, as SEP was only available as of 2014

We also assessed trends over time by socio-economic position. As SEP was available as of 2014, these trends are available for 2014–2018. When we assessed trends over time by socio-economic position, we found that the changes in diabetes prevalence were not statistically significant for any of the SEP categories except for a slight increase in the lowest SEP (P_trend_ = 0.05) (Fig. 2a). There were no statistically significant changes in diabetes clinic visits prevalence in any SEP categories (Fig. 2b). HbA1C testing increased in all SEPs, except for SEP 4, in which it decreased. However, the change was only statistically significant in SEP 2 (P_trend_ = 0.01) (Fig. 2c). HbA1c decreased across all SEP categories, however the decrease was only statistically significant in children from SEP 3 (P_trend_ = 0.02) (Fig. 2d).

## Discussion

We found substantial socioeconomic disparities in the odds of HbA1c testing as well as the odds of having poor glycemic control. While children in higher socioeconomic positions were less likely to be tested, they were considerably less likely to have poor glycemic control. Diabetes prevalence has increased slightly over time among older children, alongside an overall increase in HbA1c testing, and an overall reduction in rates of poor glycemic control. Despite the improvement in the rate of poor glycemic control, the rate remains substantially higher than the rate of uncontrolled diabetes in adults which is around 10% [18].

Our findings regarding an increased rate of poor glycemic control among children aged 10–14, are similar to those found in other studies [19, 20]. Several reasons have been suggested for the increased rate of poor glycemic control during puberty, including a reduction in insulin-stimulated glucose metabolism among pubertal children, as well as behavioral changes, such as parents becoming less involved in the diabetes management of their children during puberty [19].

Low socioeconomic status is associated with poor glycemic control in children with T1DM, even among children with health insurance [7–9, 21, 22]. Several reasons have been suggested for this association, including the burden of out-of-pockets expenses that occur even among insured individuals, co-payments and lost wages, as well as additional barriers in the access to specialist care [22]. In addition lower SEP is associated with poorer self-management behaviors which may lead to poor glycemic control [23]. In Israel, there is universal health coverage under the National Health Insurance (NHI) law, and all residents are entitled to the same access to care [13]. However, even in countries that provide universal coverage, the use of health-services differs between people with less income and education compared to those who are wealthier and better educated [24]. Our findings indicate that while children from lower SEPs are less likely to visit a specialized pediatric diabetes clinic, they are more likely to have their HbA1c levels monitored. Therefore, it is unlikely that the poor glycemic control that we found among children in the lowest SEP is due to a barrier in the access to care. It is time-consuming and difficult to obtain good glycemic control in children with diabetes, and families with financial burdens may not be able to dedicate the time and resources necessary to do so. In addition, although all residents are entitled to care under the National Health Insurance Law, specialized pediatric diabetes clinics are not distributed uniformly throughout Israel [25]. Children in peripheral areas may face substantial additional burdens when trying to access these clinics. Programs that include additional support for families from lower SEPs, may be helpful in improving the rate of good glycemic control [20]. Such support may include frequent contact from both medical and nursing staff, allowing rapid troubleshooting when issues arise regarding changes in insulin regimen.

The T1DM prevalence rate found in our study is slightly lower than the most recently reported prevalence rates in countries such as the Netherlands (0.17%), the UK (0.20%), and the US (0.19%) [26–28]. However, the ascertainment of T1DM varies in different studies. Increasing trends in the incidence and prevalence of T1DM have been reported [26, 27, 29]. In recent years, several countries have reported a halt in this increase [4–6]. Our study found a slow increase from 0.10% in 2011 to 0.12% in 2014, no change in T1DM prevalence through 2017, and an additional increase to 0.13% in 2018. Data from Israel’s National Registry of Incident T1DM cases from 2015 indicate that there was a continuous increase in the incidence of childhood T1DM cases [16]. Longer follow up time is necessary to better understand whether our results indicate the beginning of a plateau or a continued albeit slow increase.

Our study has several strengths. First, the data in our study includes all Israeli children aged 2–17 years between 2011 and 2018, and as such our study was not prone to selection biases or sampling errors. The quality of the data provided by the QICH program is exceptionally high. Data are checked at three levels. First there is an internal audit conducted by each HMO. Then the QICH program directorate performs a data audit, and finally there is an external process audit to ensure the validity of the procedures involved. Our study has several limitations which should be considered. First, we only have detailed area-level SEP information as of 2014, and we therefore only had limited follow up time to assess changes over time in the effect of SEP on diabetes prevalence and quality of care. Furthermore, we did not have information regarding additional factors that are known to influence glycemic control including duration of diabetes, ethnicity, and parental knowledge regarding diabetes. Finally, as we did not have individual-level data, we were unable to assess whether children who regularly visited a specialized diabetes clinic did in fact have better glycemic control compared to children who did not.

Based on data from multiple countries and continents – the adolescent period is characterized, in general, by poor metabolic control in patients with T1DM [19, 20, 22, 23]. Our data allows the identification of those who succeed in managing their disease well during this vulnerable period– in order to highlight the unique qualities typical of these patients. Gaining such insights may allow the identification of tools to promote better diabetes care for adolescents. Various factors may contribute to the increased rate of poor glycemic control among children from low SEP, including financial barriers to care, limited health education, and poor family communication skills. In order to further understand the causes of these disparities, additional research, using individual level data, is necessary to assess the association between specialized clinics visits and glycemic control, the associations between SEP and insulin pump therapy, and the association of the latter with clinical outcomes. These may be helpful in the planning and implementation of targeted interventions, that will close the gaps in T1DM care in children.

Additionally, in order to get a better understanding of the factors behind these disparities, qualitative research can be used. Focus groups with children with T1DM and their parents, physicians, and other caregivers will enable deepening the understanding regarding barriers to high quality care. Potential barriers include access barriers (such as taking time off work for appointments, cost and complexity of transportation to appointments), language and cultural barriers, as well as health literacy and family-provider communication concerns.

The policy recommendations may vary depending on the outcome of the above-mentioned research. If a lack of family communication is found to play a major role, interventions aimed at improving parent-adolescent teamwork in diabetes management tasks have been found to be beneficial in improving glycemic control while reducing family conflict [30, 31]. Some of these interventions can be integrated into routine diabetes clinic visits. In order to improve family-provider communication, family-centered rounds may improve communication and shared decision-making between physicians and families [32]. Family-centered care is defined as interdisciplinary care in which professionals from a variety of disciplines work collaboratively to develop a unified care plan, with active participation from patients and their families [33]. If access barriers are found to be substantial, financial incentives for both parents and youth to promote adherence to intervention programs were found to substantially improve retention [31]. Small sums provided frequently were found to be most effective [34]. Finally, incentives to HMO’s for improving health outcomes, may yield innovative strategies for screening and identifying T1DM patients who are at high risk for nonadherence and adopting personalized interventions which will improve their health outcomes.

## Data Availability

The datasets analyzed during the current study are not publicly available due to legal restrictions but are available from the corresponding author on reasonable request.
